# Known unknowns: Filling the gaps in scientific knowledge production in the Caatinga

**DOI:** 10.1371/journal.pone.0219359

**Published:** 2019-07-03

**Authors:** Thainá Lessa, Janisson W. dos Santos, Ricardo A. Correia, Richard J. Ladle, Ana C. M. Malhado

**Affiliations:** 1 Institute of Biological Sciences and Health, Federal University of Alagoas, Campus A. C. Simões, Av. Lourival Melo Mota, s/n Tabuleiro dos Martins, Maceió, AL, Brazil; 2 DBIO & CESAM-Centre for Environmental and Marine Studies, University of Aveiro, Aveiro, Portugal; National Taiwan University, TAIWAN

## Abstract

The Caatinga is an ecologically unique semi-arid region of northeast Brazil characterized by high levels of endemism and severe anthropogenic threats from agricultural development and climate change. It is also one of the least known biomes in Brazil due to a combination of inadequate investment, low regional research capacity and difficult working conditions. However, while the lack of scientific knowledge of the Caatinga is well known, the spatial and temporal distribution of knowledge production has not been investigated. This is important because such biases undermine the development of effective conservation policy and practice and increase the uncertainty associated with conservation actions. Here, we map the geography of conservation knowledge production in the Caatinga and use an innovative hurdle model to identify the presumptive factors driving these patterns. Our analysis revealed strong geographic patterns, with research sites concentrated in the east of the region and in areas close to roads and research centres. There was also a positive association between conservation knowledge production and risk of desertification, indicating that conservation scientists are responding to conservation challenges faced by Caatinga’s fauna and flora arising from climate change. Our results also highlight the pivotal role of pioneer scientists (those who develop research sites in previously unstudied/understudied areas) in determining the future geographic patterns of knowledge production. We conclude our article with a brief discussion of potential policies for increasing the spatial representativeness of conservation research in this remarkable ecosystem.

## Introduction

The Caatinga is the New World’s largest and most ecologically diverse seasonally dry tropical forest (SDTF). Situated in the interior of northeastern Brazil, the Caatinga—a Tupi word meaning “white forest” due to the pale colour of the vegetation during the frequent droughts—spreads across an area of 912,529 km^2^ and is made up of at least 135 geo-environmental units and nine distinctive ecoregions [1]. In their recent book on the ecology and conservation of this complex socio-ecological system, Silva and his colleagues [2] document high levels of endemism among the Caatinga biota, especially the 386 fish species of which more than half cannot be found in any other region of the world. This unique ecology is currently threatened by a range of anthropogenic processes associated with the 28.6 million people that inhabit the region. Indeed, agricultural development has resulted in rapid and wide-spread land-use change (more than 60% of the Caatinga has already been significantly modified by human activities) which, in turn, is causing growing problems with desertification. Moreover, this particular problem may get worse because semi-arid regions in general, and the Caatinga in particular [3], are especially vulnerable to the effects of anthropogenic climate change [4].

Despite its large size, exceptional biological and socio-cultural importance [5], ecological distinctiveness and high levels of threat, the Caatinga is one of the least studied ecological regions of the world [2, 6, 7], with knowledge shortfalls for nearly all major taxa, especially fish [8], amphibians [9] and mammals [10]. These general shortfalls are reflected in a very low biodiversity-survey effort and far fewer dedicated research teams than the adjacent and much better known humid forest ecosystems of Amazonia and the Atlantic Forest [7]. A recent study documented that 40% of the Caatinga’s plant species have never been sampled in site-based surveys, and that most recorded species only have records from a single site [11].

Quantifying and understanding conservation knowledge shortfalls are important for several reasons [12]. First, to identify spatial mismatches between scientific knowledge and conservation need [13]. For example, the Caatinga contains several areas that are undergoing rapid desertification as a consequence of habitat modification and climate change [14], with uncertain consequences for biodiversity and ecosystem functioning [2]. In addition to the formal scientific knowledge contained in academic papers, the first-hand experiences of researchers, their social networks and practical knowledge of sampling sites is also an important conservation resource that is intimately linked to scientific production [15]. Second, scientific knowledge is not created in a vacuum, being generated incrementally with new studies building on the foundations of (and partially dependent on) previous work [16]. This is especially true in ecology and conservation, where baseline data and contextual/practical knowledge of field sites and populations are critical components when planning future studies and reporting the results to the scientific community. This is reflected in the finding that new ecological research sites in the Amazon tend to be located near existing sites [15] and that the scientific productivity of protected areas is strongly associated with years since the first article based in the area was published [17]. More specifically, detailed ecological knowledge is needed to parameterize ecological niche models, key tools in our attempts to understand how species responded to past changes and to predict how they might cope with future climatic changes [18–20]. Thirdly, the presence of researchers in an area may have a safeguarding effect, independent of the research they are performing [21, 22]. Finally, understanding the factors that may drive the geography of conservation science research is a prerequisite for developing policy incentives or other measures to make research more representative and responsive to conservation needs.

There are many potential factors that may have contributed to the relative lack of ecological/conservation research in the Caatinga. For example, there are considerable practical difficulties of working in remote semi-arid habitats where the climate and terrain can make field work extremely challenging [23]. Accessibility is frequently a problem since large areas of the biome cannot be reached by road. Even when road access is available, many areas become inaccessible when it rains due to the emergence of seasonal rivers [23, 24]. It is well known that botanical samples are geographically associated with larger cities, major rivers, roads and proximity to research centres [25–28]. Another factor potentially constraining knowledge production in the Caatinga is the historical lack of research infrastructure as, until the beginning of the 21^st^ century, most Brazilian universities were concentrated in major metropolitan areas, mostly in the southern regions of the country [29]. It is thus probable that research efforts are concentrated close to highways and large cities, and the interior and local regions of difficult access are poorly sampled, as is the case in other regions [30–33].

The present work therefore has two main objectives: i) to map the geography of conservation scientific knowledge production in the semi-arid Caatinga region of northeast Brazil, and; ii) to identify the main factors that relate to conservation scientist’s choice of research site. Specifically, we use an innovative hurdle model for zero-inflated count data to examine the associations between conservation science research production research and factors related to practical convenience (e.g. accessibility, distance to universities), perceived conservation need (e.g. protected areas, desertification risk) and a history of research (years since first paper was published). Our work is inspired by the increasing body of work dedicated to characterize existing biases in knowledge about species distributions, but takes an innovative approach by mapping the location of study sites [15, 34, 35] rather than species occurrences. By focusing on a wide diversity of conservation research (as opposed to biological records), we aim to identify areas where research infrastructure is in place and where scientists have gained practical and logistic experience that will facilitate future research.

## Methods

### Data collection

We searched the scientific literature for biodiversity and conservation studies in the Caatinga using two electronic databases: Web of Science and Scielo (an online database that indexes journals from Latin America and the Caribbean, including many Portuguese-language Brazilian journals). Searches were carried out to identify papers containing the terms “Caatinga” and either “Conservation” or “Biodiversity” (in English or Portuguese) in the Title, Abstract or Keyword sections. Searches were initially carried out in October 2014 and again in January 2018 to include the most recent publications up to the end of 2017. Electronic copies of each paper were downloaded whenever possible and their content was screened to confirm its relevance for our study. Manuscripts were rejected if the research was not carried out in the Caatinga or the study topic was clearly unrelated to conservation or biodiversity.

For each identified paper, we extracted information regarding: i) year of publication, ii) research institution of the lead (first author) researchers, and iii) *taxon* studied (at class level) (S1 Appendix). We extracted from the Web of Science Brazil’s temporal data about biodiversity and conservation papers to analyse the proportion of Caatinga scientific production in national terms. This search was carried out using “conservation” and “biodiversity” as keywords (same as above) and selecting all papers with an institutional address in Brazil.

Locality and geographic coordinates of the research site(s) was identified for each study with the objective of mapping the distribution of research sites and highlighting regions of low knowledge production. When the manuscript did not provide point locations, we identified the location of the study site(s) through contextual information, following a process similar to the one described in dos Santos et al. [15]. More specifically, this information often described a nearby settlement or town, for which the centroid was mapped using Google Earth. We used QGis 2.6 to create maps of knowledge production at the scale of 25km^2^ cells. We identified the quantity of study sites and the year of the first recorded study by count of point locations per grid. For visualization purposes we organized grid cells based on the number of studies recorded: from knowledge gaps (n = 0) to well-studied areas (n > 25).

### Data analysis

The response variable in our study was the number of identified study sites for each 25 km^2^ grid cell in the Caatinga region. To explore the factors potentially driving the distribution of study sites, we considered a broad set of explanatory variables (Table 1) that encompass practical convenience, perceived conservation needs and history of conservation science research to assess the key factors that could affect the conservation science knowledge production across the Caatinga. This is because the factors driving a scientist’s decision to work in any given research site (e.g. in a protected area, a desertification zone, close to a university, etc.) will inevitably vary between individual researchers and projects.

**Table 1:** Definition and justification of response and explanatory variables entering the hurdle model. Each variable was characterized for every 25km^2^ grid cell considered in this study (see Fig 1).

Variable	Format	Units	Justification	Source	Summary statistics
Study sites*	Continuous	Number of study sites per grid cell	The number of study sites is indicative of conservation research effort in a given area.	Scientific papers	Avg: 0.93 Min: 0 Max: 33
Human Population Density	Continuous	Number of persons per square kilometre	The presence of a human population is predicted to be associated with research infrastructure (e.g. accommodation), accessibility (e.g. roads, bridges, etc.) and local knowledge.	Gridded Population of the World, v4	Avg: 686 Min: 0 Max: 42964
Road Density	Continuous	Road length (km) per square kilometre	Several studies have shown that ecological research and collecting clusters near roads since it is more convenient to place research sites in accessible areas.	Calculated with data from Open Street Map	Avg: 0.02 Min: 0 Max: 0.38
Years since first publication	Continuous	Number of years since first recorded publication until 2018	Science is iterative, with previous studies often providing the basis for future research. Moreover, established field sites may be more convenient and secure than new sites.	Web of Science and Scielo	Avg: 6.2 Min: 1 Max: 25
Distance to University	Continuous	Distance in km from cell centroid to nearest university	Research sites based near universities are both more convenient in terms of time spent travelling and, potentially, less costly.	Calculated with data from Google Maps	Avg: 29.9 Min: 7.9 Max: 98.4
Protection Status	Categorical	Protection status: Full protection; Sustainable use; Unprotected	Protected areas are key foci for biodiversity conservation and often contain better preserved ecosystems and basic research infrastructure.	Brazilian Ministry of the Environment	-
Desertification Susceptibility	Categorical	Desertification susceptibility classes: Extreme; Very severe; Severe; Moderate; None	Desertification is one of the main anthropogenic threats to biodiversity in the Caatinga and there is therefore an urgent need for research on its causes and ecological consequences.	Brazilian Ministry of the Environment	-

The response variable is highlighted with an asterisk (*).

These variables were used to model conservation science effort in the Caatinga using hurdle models for zero-inflated count data [36]. This choice of modelling approach is due to our data containing a large number of grid cells (n = 808) with no conservation studies recorded. Hurdle models are composed of two parts: a hurdle component, which models the presence or absence of research in a cell, and a zero-truncated count model which models the number of research outputs for cells with at least one recorded research product.

Furthermore, the choice of research site is a complex decision-making process that is unlikely to be accurately represented by a single model, and we therefore adopted a multi-model inference approach. Such an approach allows us to identify the most parsimonious models regarding the relative importance of each explanatory variable [37] and to carry out a weighted-average estimate of the effect of different explanatory variables in the decision process [38, 39]. Based on our hypotheses about which variables may influence the presence and abundance of research (Table 1), we calculated all possible model combinations (without interactions) relating the presence and number of scientific articles to our explanatory variables. Prior to modelling, we tested the correlation between variables and found no evidence of severe multicollinearity (Pearson’s *r*<0.70 for all variables). We then identified plausible models according to Akaike’s Information Criterion corrected for small sample size (AICc) and considered all models with ΔAICc ≤ 4 in relation to the most parsimonious model (S1 Table) for a full model averaging process [37].

All analyses were carried out with R Statistical Software [40]. Hurdle models were implemented using the function ‘hurdle’ of the package ‘pscl’ [36] and multi-model inference and averaging were carried out with package ‘MuMIn’ [41].

## Results

**Fig 1:**
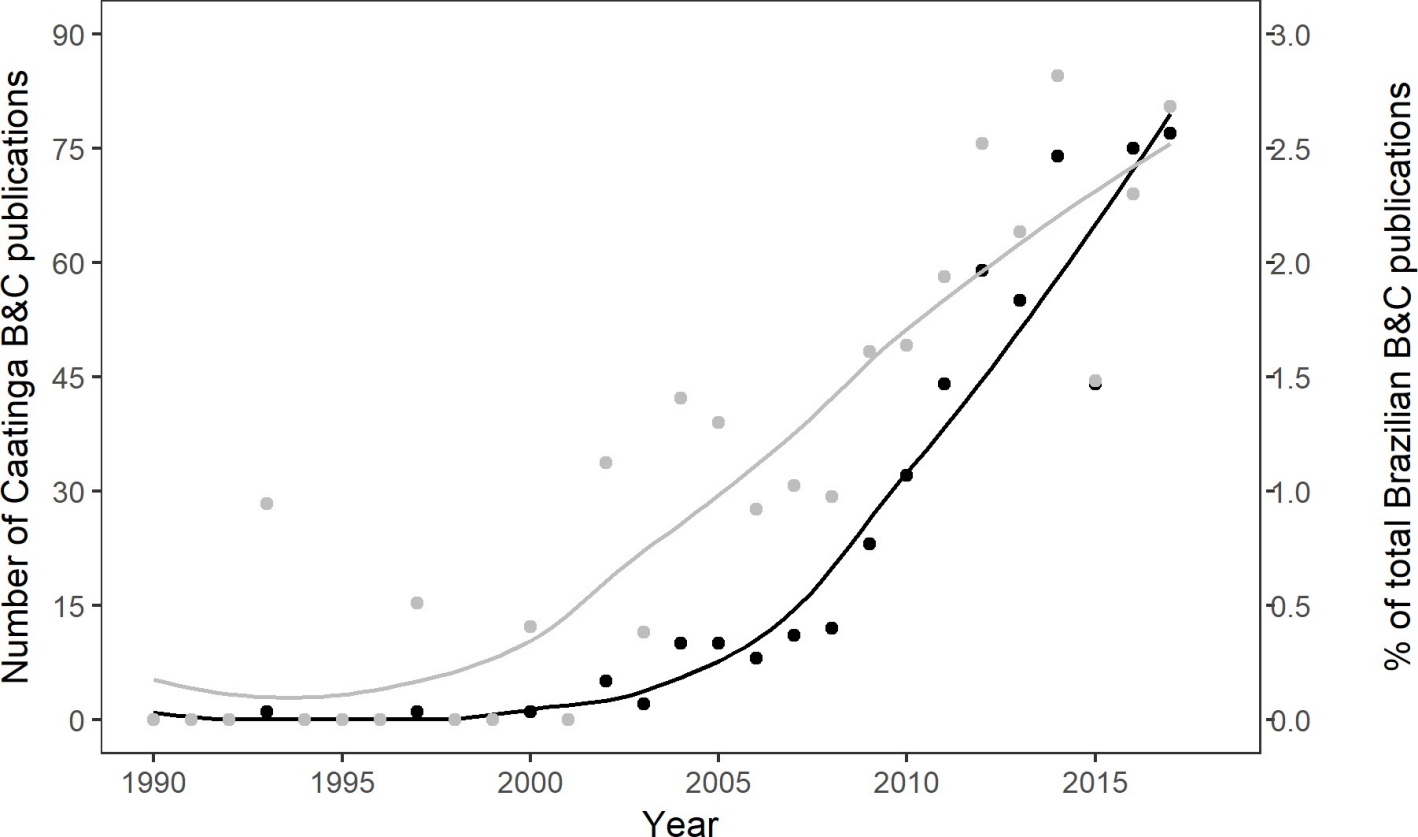
**Number of Caatinga biodiversity and conservation publications (in grey) and percentage of Caatinga publications in relation to Brazilian biodiversity and conservation research (in black)**.

We identified 785 scientific papers using our search methodology and extracted information from 544 papers that fit our search criteria and were available for download. The oldest paper identified with a focus on biodiversity and conservation in the Caatinga in our database was from 1993. While a few more studies were published in the following decade, the majority were published after 2008. Specifically, scientific manuscripts published from 2008 onwards represent 90.9% (n = 495) of papers in our database. Knowledge production was positively correlated (Pearson’s r = 0.96) with production of scientific articles on conservation and biodiversity for the whole of Brazil (data from Web of Science), although the proportion of Brazilian conservation research dedicated to the Caatinga has nevertheless increased over the last few years (Fig 1).

**Fig 2:**
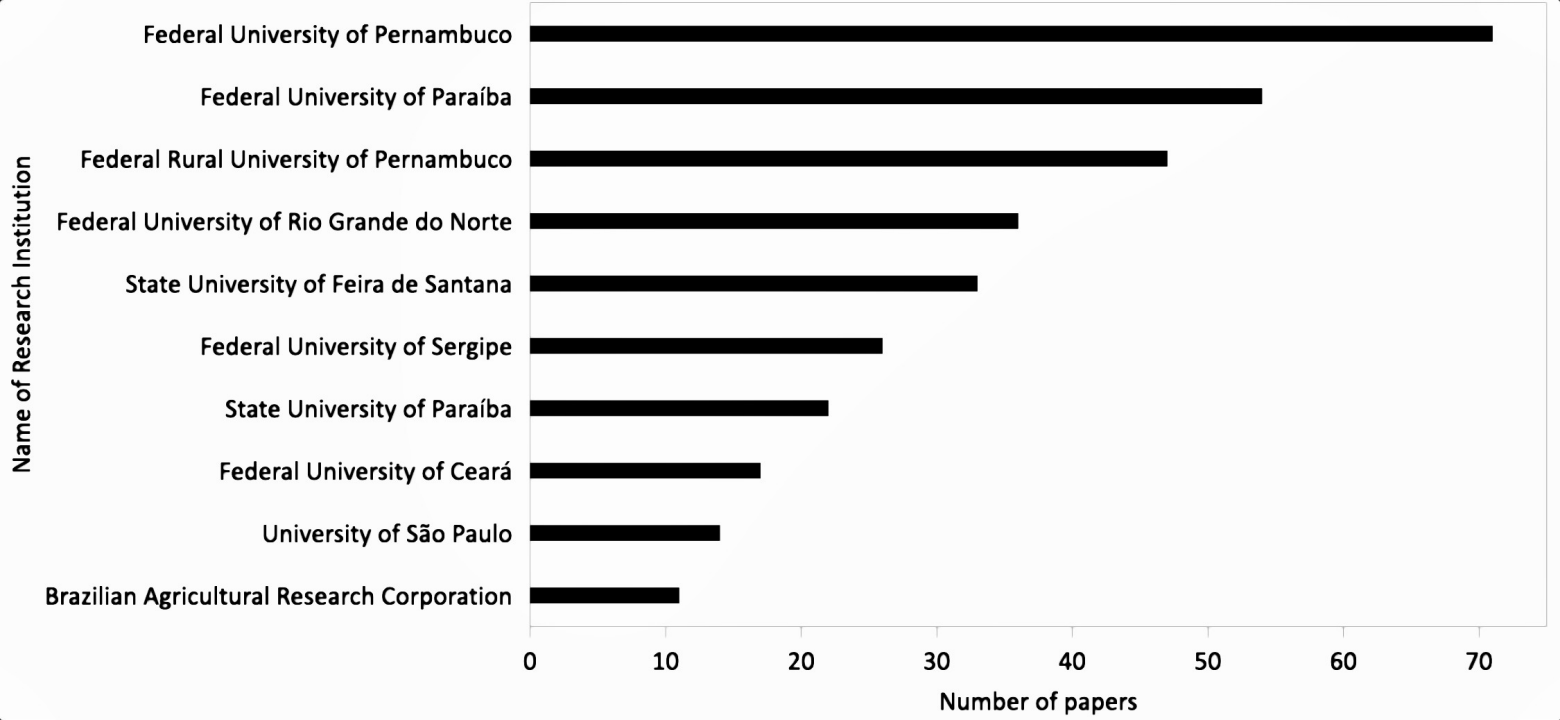
Top-10 contributing universities in number of scientific publications for Caatinga biodiversity and conservation science.

Researchers associated with foreign research institutions were responsible for some of the earliest studies. However, the ten highest contributing research institutions (Fig 2) in terms of scientific outputs were Brazilian and responsible for more than 60% of identified studies. These institutions include the Federal University of Pernambuco, the Federal University of Paraíba and the Federal Rural University of Pernambuco, and each of them published at least 10 scientific papers. The majority (54.7%) of research institutions in our database contributed with only a single publication.

Papers with a focus on plants (n = 191) were more represented than those about vertebrates (n = 115, mammals = 42, birds = 31, reptiles = 23, amphibians = 19) or invertebrates (n = 76). Ethnological studies were also well represented (n = 45). Despite very high endemicity and high threat level due to their vulnerability to changing climatic conditions, fish (n = 14) were the least represented taxonomic group in our database.

**Fig 3:**
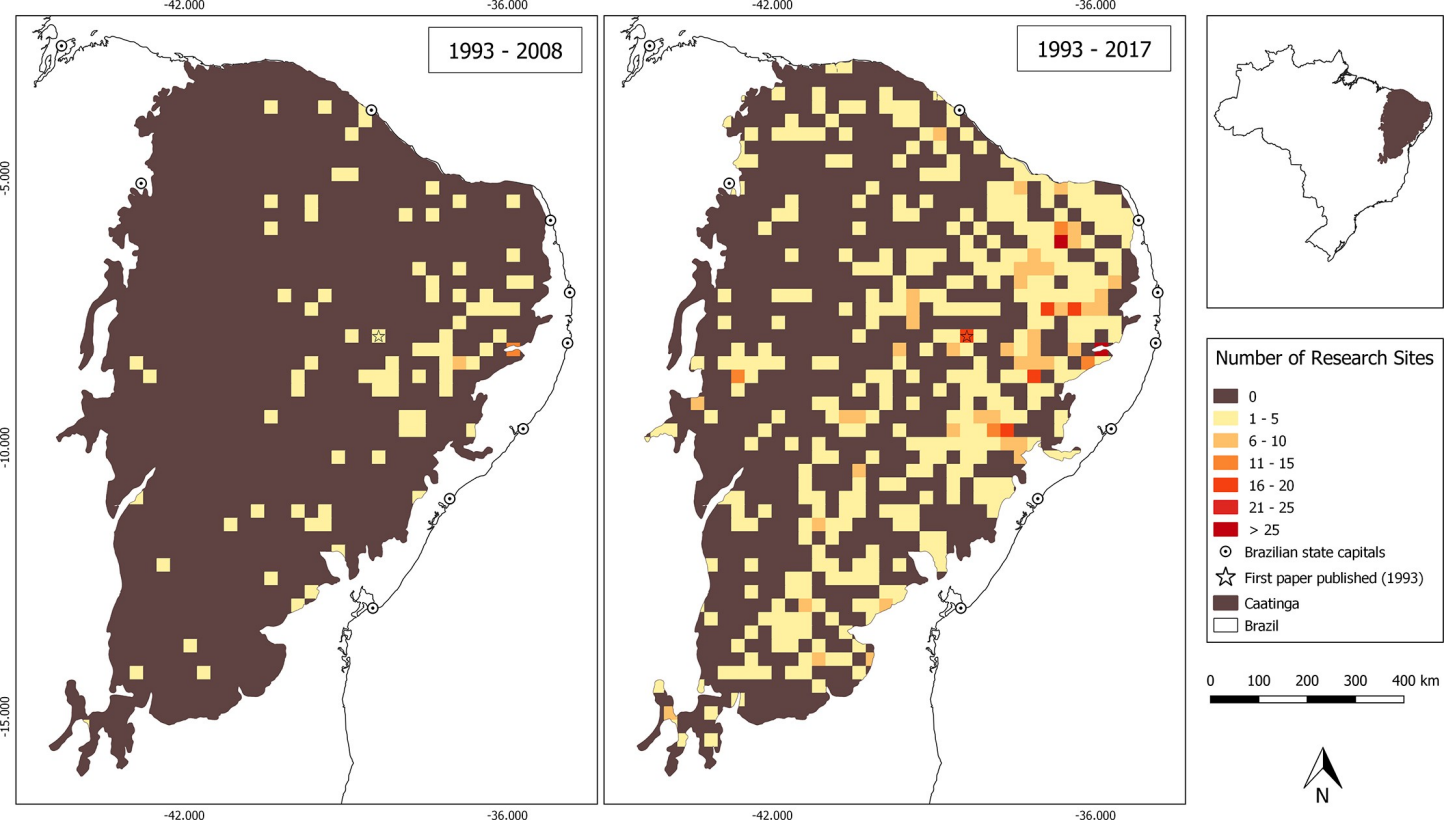
**Distribution of recorded field sites for Caatinga biodiversity and conservation research between 1993 and 2008 (left panel) and 1993 and 2017 (right panel)**.

A total of 1,146 geographic coordinates were collected from 544 studies, 25.6% of which were centroid locations derived from contextual information (site names, town names, etc). After mapping studies onto 25km^2^ grid cells, our results suggest that approximately 65.7% of the region has not been the subject of peer-reviewed research (n = 0) and a further 29.8% recorded a low number of conservation studies (n = 1–5) (Fig 3). There is a pronounced concentration of research effort in the north-eastern and eastern parts of the Caatinga, with research effort concentrated near the limits of the ecoregion and close to the capitals of the north-eastern states (e.g. Salvador in Bahia, Aracaju in Sergipe, Maceió in Alagoas), where a large part of the population and research centres are concentrated. Correspondingly, the centre, west and northwest of the Caatinga are characterized by lower levels of scientific research.

Our modelling analysis (Table 2; S1 Table) indicated that the presence of research sites was positively associated with road density (relative importance = 0.95) and negatively associated with distance from universities (relative importance = 1.00). Interestingly, while land protection status showed no relevant association (relative importance = 0.32) with the presence of research in a given area, there was a significantly higher probability of research being carried out in areas under severe or very severe risk of desertification (relative importance = 1.00).

**Table 2:** Results of Hurdle modelling analysis. The hurdle component refers to presence/absence of research sites; the count component refers to number of research sites in cells with at least one recorded study.

Explanatory variable		Hurdle component		Count component	
		Estimate ± SE	Rel. import.	Estimate ± SE	Rel. import.
Intercept		-0.90 ± 0.10*	-	-1.48 ± 0.30*	-
Distance to university		-0.36 ± 0.07*	1.00	-0.20 ± 0.09*	0.91
Population density		0.06 ± 0.08	0.27	0.07 ± 0.06	0.45
Protection status	Sustainable use	0.24 ± 0.19	0.32	0.55 ± 0.22*	1.00
	Full protection	0.39 ± 0.29	-	0.84 ± 0.32*	-
Road density		0.17 ± 0.08*	0.95	0.04 ± 0.08	0.24
Desertification susceptibility	Moderate	0.02 ± 0.15	1.00	0.05 ± 0.20	1.00
	Severe	0.84 ± 0.22*	-	0.50 ± 0.25*	-
	Very severe	1.13 ± 0.22*	-	1.12 ± 0.22*	-
	Extreme	0.81 ± 0.48	-	0.44 ± 0.51	-
Years since first publication		-	-	0.83 ± 0.10*	1.00
Log (theta)		-	-	-0.46 ± 0.33	-

Significant effects are highlighted with an asterisk (*: p-value <0.05).

A slightly different pattern emerges when considering the number of studies; both land protection status (relative importance = 1.00) and areas at risk of desertification (relative importance = 1.00) were associated with significantly higher volumes of research. The number of conservation studies in the Caatinga was also significantly and positively associated with the age of first publication (relative importance = 1.00) and distance to the nearest university (relative importance = 0.91).

## Discussion

Conservation science is a broad area that covers many different sub-disciplines, from ecology to sociology [42]. Many of these studies are based on collecting data from field sites with varying levels of human influence, with a typical focus on ‘natural’ areas with low levels of human habitation and largely intact ecological communities. Such areas often host the remnant populations of rare and endangered species, further increasing their value as foci for conservation research. However, such sites are often far from the urban centres where scientists live and work and therefore may not be practically convenient for many types of experimental and observational research. Moreover, if an area has never been used for scientific research there may be: i) uncertainty over its appropriateness for a given study, and; ii) a lack of contextual and baseline information that can be used to inform the design of a new study. As a consequence of these two opposing forces (practical convenience and conservation need), there are frequent mismatches between the actual and ideal locations of study sites and identity of study organisms [43].

We reasoned that such mismatches are likely to be especially pronounced in the Caatinga of northeast Brazil due to the extremely difficult (for humans) working conditions, with daytime temperatures often reaching 40°C and a native flora characterized by a spectacular array of thorns, spines and hooks [23]–adaptations against the now extinct megafauna [44]. Our results largely confirmed this, indicating that conservation research about the Caatinga is relatively scarce and patchily distributed. This is unlikely to be due to biases in our dataset since previous studies have also noted the relative paucity of scientific studies in the region [2, 6], a factor that may negatively contribute to the implementation of effective conservation policy. Nevertheless, it is important to acknowledge that our database is by no means comprehensive: some relevant articles were probably not retrieved because they were not recognized as conservation articles using our simplified search strategy or as having been conducted in the Caatinga if explicit mentions to this ecoregion are omitted. However, this should not lead to any systematic biases (spatial or temporal) in our dataset and should not therefore significantly diminish the robustness of our conclusions.

Predictably, most articles in our database were of recent origin: this concords with recent work in Amazon [15] and is indicative of both research trends in the neotropics [45] and the relatively recent origins of conservation biology [42, 46]. Indeed, one of our search terms, “biodiversity”, is a neologism coined in 1986, and only coming into common usage in the 1990s [47]. There are certainly many older articles that, while relevant to conservation, did not fit our search criteria and therefore did not appear in the database–a good example would be Andrade-Lima’s [24] early treatment of the plant communities of the Caatinga. Another interesting aspect of the recent development of conservation science in the Caatinga is that it has largely been led by Brazilian scientists, unlike what has happened in other regions of Brazil [48]. While foreign researchers were responsible for some of the earliest studies in our database, which may have provided important scientific foundations for research in this region, our data shows that the large majority of recent publications are native-led. This suggests that lack of foreign interest and local research capacity may have slowed the development of Caatinga conservation research, and that the development of research capacity in the region during the beginning of the 21^st^ century may have played a key role in recent developments [49]. This assessment is supported by data suggesting that Brazilian scientific output nearly doubled between 2005 and 2009 [50], and the boost in research outputs during this period may also justify the observed growth in Caatinga research from 2008 onwards.

Our data clearly reveal strong geographical patterns of conservation research, probably linked to three main factors. The first is the logistics of field-based studies, as reflected in the significant association between road density and the presence/absence of research in an area. Known as “road-side bias”, this effect is well known in studies of biological collections [31] and is primarily caused by the difficulties of establishing and maintaining field sites and experiments while carrying everything on foot. Due to the rocky geomorphology and dense vegetation of the Caatinga, even off-road vehicles may have difficulty entering more remote areas. There was also a negative effect of distance to university research centres, suggesting that researchers (understandably) tend to choose research sites close to their place of work. The second factor is perceived conservation need. Interestingly, there was a positive association between the presence and volume of conservation research and areas at high risk of desertification. This indicates that researchers are engaging with this important anthropogenic driver of biodiversity loss and conducting their research in the areas that may be most impacted. A third factor is historical precedent. As documented in previous studies [15, 17], we found a strong relationship between the volume of scientific research and the time that had elapsed since the first study in that area had been published. This highlights the importance of pioneer research, providing both baseline data and establishing new study sites that can be used repeatedly. This finding also demonstrates the potentially strong social aspect of scientific research, whereby younger researchers may be influenced in their choice of sampling location and research theme by their supervisors and mentors.

Interestingly, protected areas were only statistically associated with the volume rather than the presence/absence of conservation research. This result is hard to interpret given the lack of preference for carrying out research within protected areas and may be linked to a relatively small number of very productive sites within protected areas. However, although the probability of research being carried out in PAs is not quite significant, the trend is going in the predicted direction. In this context it is perhaps relevant that the Caatinga is one of the Brazilian biomes with the greatest number of Ecological Stations—protected areas with an explicit remit to host scientific research [51, 52]. These areas have played a major role in generating ecological baselines and, though typically encompassing relatively small areas, may have been important in driving the observed associations. In a more general context, the lack of a strong association between research presence and land protection implies that many of the Caatinga’s PAs are being underutilized as a scientific resource, particularly sustainable use areas. This merits further attention since research in these areas has the potential to positively contribute towards the sustainable use of the biome.

Finally, it is important to recognize the limitations of our model. As with any other attempt to formally identify and quantify the drivers of a complex, multifaceted spatial phenomenon (conservation research effort), our sub-set of explanatory variables is almost certainly incomplete. For example, targeted research funding may have led to greater research effort in certain states or municipalities. In this case, we did not include this factor in our model because the data is either unavailable or incomplete. A second potential limitation is, to our best knowledge, the current inability of zero-hurdle models to explicitly consider a spatial correlation structure as a means to account for spatial autocorrelation in the data. While it is likely that our data shows a degree of spatial autocorrelation, this is likely to be driven by factors such as accessibility and proximity to urban centres. We attempted to account for this by explicitly including explanatory variables that are likely to cause spatial clustering (e.g. road density, distance to universities) but this issue warrants further exploration as new spatial datasets and analytical methods become available.

In summary, our research reveals a complex picture of conservation research in the Caatinga where, despite dramatic recent increases in the volume of published studies, there remain large areas with little or no research presence. Diminishing the number and extent of these research ‘cold spots’ is not straightforward, although there are several policy approaches that could help, such as: i) providing financial incentives to work in western regions that might harbour high levels of endemics and species unknown to science; ii) investment in basic research infrastructure (e.g. accommodation quarters, laboratories, small offices), especially in protected areas, and; iii) providing support and incentives for pioneer researchers who are prepared to work on potentially risky projects and open up new areas to research. Much of this early research may be focused on establishing baselines and other descriptive studies that are not easily published in peer review journals and may therefore need to be funded through non-standard channels.

## Supporting information

S1 Appendix Database including information of papers and their geographic coordinates, and variables description. (XLSX)

S1 Table Summary table of the zero hurdle count models relating ESEC scientific productivity to our set of explanatory variables. (DOCX)
