# Supplementary material for: Known unknowns: Filling the gaps in scientific knowledge production in the Caatinga
Source: PLoS One. 2019 Jul 3;14(7):e0219359. doi: 10.1371/journal.pone.0219359 (PMC6608954; doi:10.1371/journal.pone.0219359)
Supplement: S1 Table — (DOCX) [file pone.0219359.s002.docx]

Supplementary Materials

S1 Table - Summary table of the zero hurdle count models relating ESEC scientific productivity to our set of explanatory variables.

| Rank | Model | Distance to university | Population density | Protection status | Road density | Desertification susceptibility | Years since first publication | AICc | ΔAICc | ωAIC |
| --- | --- | --- | --- | --- | --- | --- | --- | --- | --- | --- |
| 1 | Zero hurdle | X |  |  | X | X |  | 2775.85 | 0.00 | 0.16 |
|  | Count | X |  | X |  | X | X |  |  |  |
| 2 | Zero hurdle | X |  |  | X | X |  | 2776.29 | 0.44 | 0.13 |
|  | Count | X | X | X |  | X | X |  |  |  |
| 3 | Zero hurdle | X |  | X | X | X |  | 2776.99 | 1.14 | 0.09 |
|  | Count | X |  | X |  | X | X |  |  |  |
| 4 | Zero hurdle | X |  | X | X | X |  | 2777.44 | 1.59 | 0.07 |
|  | Count | X | X | X |  | X | X |  |  |  |
| 5 | Zero hurdle | X |  |  | X | X |  | 2777.47 | 1.62 | 0.07 |
|  | Count | X |  | X | X | X | X |  |  |  |
| 6 | Zero hurdle | X | X |  | X | X |  | 2777.65 | 1.80 | 0.07 |
|  | Count | X |  | X |  | X | X |  |  |  |
| 7 | Zero hurdle | X | X |  | X | X |  | 2778.09 | 2.24 | 0.05 |
|  | Count | X | X | X |  | X | X |  |  |  |
| 8 | Zero hurdle | X |  |  | X | X |  | 2778.34 | 2.49 | 0.05 |
|  | Count | X | X | X | X | X | X |  |  |  |
| 9 | Zero hurdle | X |  |  | X | X |  | 2778.53 | 2.68 | 0.04 |
|  | Count |  | X | X |  | X | X |  |  |  |
| 10 | Zero hurdle | X |  | X | X | X |  | 2778.62 | 2.77 | 0.04 |
|  | Count | X |  | X | X | X | X |  |  |  |
| 11 | Zero hurdle | X | X | X | X | X |  | 2778.79 | 2.94 | 0.04 |
|  | Count | X |  | X |  | X | X |  |  |  |
| 12 | Zero hurdle | X | X |  |  | X |  | 2779.20 | 3.35 | 0.03 |
|  | Count | X |  | X |  | X | X |  |  |  |
| 13 | Zero hurdle | X | X | X | X | X |  | 2779.24 | 3.39 | 0.03 |
|  | Count | X | X | X |  | X | X |  |  |  |
| 14 | Zero hurdle | X | X |  | X | X |  | 2779.27 | 3.42 | 0.03 |
|  | Count | X |  | X | X | X | X |  |  |  |
| 15 | Zero hurdle | X |  | X | X | X |  | 2779.49 | 3.64 | 0.03 |
|  | Count | X | X | X | X | X | X |  |  |  |
| 16 | Zero hurdle | X | X |  |  | X |  | 2779.64 | 3.79 | 0.02 |
|  | Count | X | X | X |  | X | X |  |  |  |
| 17 | Zero hurdle | X |  | X | X | X |  | 2779.68 | 3.82 | 0.02 |
|  | Count |  | X | X |  | X | X |  |  |  |
| 18 | Zero hurdle | X |  |  | X | X |  | 2779.78 | 3.93 | 0.02 |
|  | Count |  |  | X | X | X | X |  |  |  |
